# Meditating musicians: investigating the experience of music students and professional musicians in a brief mindfulness course to address music performance anxiety

**DOI:** 10.3389/fpsyg.2025.1567988

**Published:** 2025-05-09

**Authors:** Serena Paese, Andrea Schiavio

**Affiliations:** School of Arts and Creative Technologies, University of York, York, United Kingdom

**Keywords:** music performance anxiety, music practice, wellbeing, mindfulness, meditation

## Abstract

Previous research shows that meditation practice helps reduce Music Performance Anxiety (MPA), positively impacting the musicians’ wellbeing and performative skills. Several meditation types have been explored, but further investigation into additional methods is warranted to provide a more comprehensive understanding of the impact and potential use of meditation to address MPA. The present work aims to provide novel insights into the perceived impact of three meditation types, namely: body-centered meditation, meditation on thoughts and affect-centered meditation. Qualitative data were collected from 12 musicians participating in two short introductions to mindfulness courses, held within a pilot study and a main case study, via diaries and open-ended responses. The findings suggest that a four-week mindfulness course can enhance wellbeing, boost emotional balance, and mitigate the occurrence of MPA.

## Introduction

Music performance anxiety (MPA) is widespread among professional musicians and students, and is typically perceived as a “disabling” problem for performance quality and general health (Cox and Kenardy, 1993; Studer et al., 2011; Steptoe and Fidler, 1987; Wesner et al., 1990). Given the potential for its onset in early music career (Kenny and Osborne, 2006), the identification of effective strategies to address MPA and improve the quality of musical performance is of utmost importance. Not only does this objective specifically address a concrete need for musicians; it also represents a key *desideratum* of educational institutions who seek to promote healthy approaches to skill acquisition and development (Gembris et al., 2018; Perkins et al., 2017; Spahn et al., 2021). As such, recent contributions have examined a wide range of practices that appear to be particularly beneficial in these contexts.

Research findings have suggested positive effects of meditation on MPA, making it a potentially suitable tool to help mitigate the phenomenon. Among the most studied meditation techniques yoga, the Mindfulness-Based Stress Reduction (MBSR) protocol (Kabat-Zinn, 2003), and Zen meditations are probably the most well-known (Chang et al., 2003; Czajkowski et al., 2020; Diaz, 2018; Khalsa et al., 2009; Stern et al., 2012). Extensive studies on mindfulness have highlighted the components of attention regulation, body awareness, emotion regulation, and change in perspective on the self (Hölzel et al., 2011). Previous studies, primarily conducted in non-musical contexts, have shed light on various cognitive mechanisms such as mindfulness, meta-awareness, self-inquiry, cognitive reappraisal, and perspective taking, which are associated with different meditation approaches (Dahl et al., 2015). However, the impact of specific meditative practices on an individual’s perception of their own music performance anxiety (MPA) remains largely unexplored in current academic research.

Traditionally, mindfulness and meditation interventions have been explored over an eight-week period, which has been the primary timeframe under investigation (Chang et al., 2003; Czajkowski et al., 2020; Khalsa et al., 2009; Stern et al., 2012). However, the exploration of shorter interventions has gained traction due to the emergence of positive effects on emotions and attention (Gorman and Green, 2016; Thompson et al., 2021). Consequently, the potential value of shorter meditation interventions is gaining attention for its applicability in designing support and development activities for students and for self-directed use. The present qualitative case study is consistent with this recent focus and addresses the dearth of qualitative and longitudinal studies on MPA in related research (Barros et al., 2022).

### Aims and study design

The present research aims to investigate the perceived impact of three principal mindfulness meditation types utilized by music students and professional musicians, while also examining the underlying cognitive and emotional mechanisms associated with these practices. The cognitive and emotional regulation mechanisms are investigated through the lens of previous theories elaborated by Dahl et al. (2015) which are exposed in the subsequent section dedicated to the deductive thematic analysis of the main case study. Driving research questions are:

1. How do music students and professional musicians perceive the impact of various mindfulness meditation practices on MPA and overall wellbeing?
2. What cognitive and emotional regulation processes are involved in these meditation practices that may help reduce MPA?

To address these questions, we conducted an exploratory case study in November and December 2022. Pre-, mid-, and post-intervention data were collected via diaries and open-ended questions, with the aim of gaining insight into the participants’ experience of individual mindfulness meditations on MPA manifestations. The study obtained ethical approval from the Arts and Humanities Ethics Committee of the University of York in 2022 and was piloted in 2021 after obtaining similar ethical approval. In the sections below, we will first present the pilot study, followed by the main case study.

## Pilot study

### Methods

#### Participants

Participants were recruited among the University of York undergraduate and postgraduate music students. The study was presented as a 21-day meditation course for music students interested in acquiring skills for counteracting anxiety in music performance. The participants were provided with comprehensive information regarding the components of the meditation intervention, including a detailed information sheet and a summary sheet. The information highlighted the benefits of integrating a daily meditation practice as an introductory step to their instrumental practice, the importance of maintaining a daily diary, and the value of attending a weekly meeting. Five students (women = 3, men = 2, age range = 19–28, mean = 22, *SD* = 3.46) completed the meditation course and the questionnaires (see Table 1). One student had previously used meditation as a tool to address MPA. All participants reported to suffer from MPA, two students reported to suffer from general anxiety beyond MPA and none of the participants took medications.

**Table 1:** Participants who completed the 21-day meditation course.

Participant	Age	Instrument	Level of study
1	27	Guitar	Postgraduate
2	20	Piano	Undergraduate
3	23	Piano	Undergraduate
4	28	Voice	Postgraduate
5	19	Voice	Undergraduate

#### Data collection

Qualitative data were gathered via a diary in which participants were encouraged to provide a detailed account of their daily meditative practice, including the duration, type of meditation, and the principal perceived effects. The diaries were distributed to participants at the outset of the study and were sent back to the researchers at the conclusion of each week via email. The objective of this approach was to ensure that participants remained engaged with the diary as a means of documenting their meditative practices, primary emotions, and observed impacts. At the conclusion of the course, participants were invited to complete an open-ended questionnaire via Qualtrics, in order to provide their initial impressions of the course and to ascertain its perceived impact on MPA.

#### Procedures

The brief meditation course was conducted online in October 2021, subsequent to the ethical approval obtained from the Arts and Humanities Ethics Committee of the University of York in September 2021. The decision to adopt an online format was made in recognition of its relative ease of management in the post-pandemic period, when a number of restrictions on in-person activities were still in effect. It was further considered an appropriate alternative (Ivtzan et al., 2016; Krusche et al., 2013). The 21-day course was taught by Julie Parker, a mindfulness practitioner based at University of York who is also a practitioner of the Alexander Technique. The three meetings followed the same structure, comprising an introduction to the meditative practice, practical exercises, post-practice reflections and a question-and-answer session. To ensure reflexivity and appropriateness of the procedures, a debriefing with the course teacher was conducted following each meeting. The meditations practiced (body-scan, 5-sense, and compassion meditation) were selected on the grounds that they are regarded as among the most frequently employed and efficacious when it comes to their application in relation to MPA (Paese and Egermann, 2024). They were also arranged in a similar order to the mindfulness protocols (MBSR; Kabat-Zinn, 1994). Table 2 offers additional details of the meditative practices undertaken.

**Table 2:** Meditation types practiced within the 21-day mindfulness intervention.

Week	Meditation
1	*Body-scan meditation*: It is the guided, sequential exploration of anatomical body parts that starts with the feet, culminates with the head and attention that includes the entire body. It is practiced lying down or seated, and aims to promote an attitude of curious, non-judgmental attention to bodily sensations (Kabat-Zinn, 1994).
2	*5-sense meditation*: The concept behind the five-sense meditation is to welcome and observe sensations without judgment, without trying to modify or analyze them. It is practiced seated, and involves conscious attention on each sense, one at a time (touch, sight, hearing, smell, taste) or in combination, to enter a state of deep mental presence (Kabat-Zinn, 1994).
3	*Compassion meditation*: It is a meditative technique with roots in the Buddhist tradition, but it can be adopted and practiced by anyone regardless of religious belief. It is aimed at connecting with one’s own and others’ suffering, to awaken the compassion that is inherent in all of us (Monaghan and Viereck, 2011; Salzberg and Kabat-Zinn, 1996).

In addition to attending the weekly meeting, participants were expected to engage in a meditation daily practice of a minimum of 10 min prior to their instrumental practice.

### Data analysis

#### Findings and discussion

The diaries and open-ended questions were analyzed with the thematic analysis as outlined by Braun and Clarke (2006). This cross-case thematic analysis yielded major insights into three areas: (i) the experience of daily meditative practice, with an examination of the difficulties encountered and the resources employed by the music students to complete the course; (ii) the perceived impact on MPA and wellbeing, with aspects related to the body and emotional awareness, and multifaceted manifestations of MPA; (iii) the observed emotional state, with fluctuations of positive, negative and ambivalent emotions that alternated throughout the weeks of meditative practice.

#### Theme 1: the experience of daily meditative practice

The regular practice of meditation necessitates perseverance and discipline (Kabat-Zinn, 1994; Monaghan and Viereck, 2011). The initial phase of establishing a routine as a new beneficial habit is often challenging for a number of reasons (Czajkowski, 2018). Music students, already versed in the concepts of assiduity and consistency from their prior instrumental training, nevertheless encountered significant challenges in this study, in adapting to the demands of meditation.

A significant challenge encountered by students was the presence of distracting elements, which rendered meditative practice arduous. These elements encompassed mental rumination, internal discourse, and physical discomfort and are captured by the following excerpts: “I had to try to not be distracted by muscular tension and focus on the meditation,” “my mind is still running at a fast pace from a lot of stimulation during the day” (participant 1); “it feels really hard to find and devote some time to meditation,” “I like the results and the effect meditation gives me, but I feel it is not right for me” (participant 2); “meditating was challenging,” “it was frustrating to bring myself back to the present as I was distracted by my thoughts” (participant 3); “I perceive my noisy mind,” “I feel some tension in my back, the muscle around my eyes, shoulders, neck” (participant 4); “many thoughts in my mind, waiting for the meditation to finish,” “I was thinking to another thing” (participant 5).

Indeed, meditation practice engenders a state of heightened self-awareness, thereby prompting a heightened sensitivity to physical sensations, emotions, and thoughts that typically persist in the background. This heightened awareness is considered one of the primary benefits of meditation. However, in the nascent stages of meditation practice, it can impede an individual’s ability to accept and manage perceptions and sensations (Kabat-Zinn, 1994).

A number of logistical issues were also identified, evidencing the challenge of identifying a suitable location for meditative practice, particularly for music students who may have limited financial resources and are often compelled to share living spaces, like for participant 2, who states: “Living with a roommate makes things even more difficult. Sometimes I think: ‘Why not meditate now?’, and then something happens -urgent business, roommate comes into the room- and I have to delay my meditation practice.” It may be of particular interest to educational facilities, as by providing sufficient study spaces, instrumental studies that incorporate practices of body and emotional awareness, and the development of beneficial skills for musical practice and performance can be ensured.

A previous study conducted with music students had already highlighted the difficulties that emerged, especially during the initial settling-in phase of attending a mindfulness course for musicians (Czajkowski, 2018). The insight emerging from the experience of students who have completed this 21-day course in mindfulness practices for musicians relates to a perceived need for enhanced care and support from the course facilitator and the educational institution itself. In the design of interventions to support students’ wellbeing and address problems associated with instrumental training, such as MPA, it would be advisable to provide constant additional support from the meditation teacher to ensure a consistent daily individual practice. In addition, the educational institution that might deliver these courses can also contribute to this support by providing more space for practice.

#### Theme 2: perceived impact on MPA and wellbeing

An increase in bodily, cognitive, and emotional awareness was identified as the core of the perceived impact on wellbeing and MPA. Indeed, the practice of body-scan and five-sense meditations resulted in students reporting heightened bodily sensations, both positive and negative. The meditative practice fostered an acceptance of these sensations, accompanied by an acquired ability to stay in the discomfort of unpleasant feelings, emotions and thoughts. Consider the following exemplary quotes: “meditation allowed me to accept my body feeling tired after a long day and led me into a good mental and physical space for sleep” (participant 1); “I am allowing my emotions to flow,” “I feel in a process of acceptance” (participant 3); “I realized I have more space inside me to breathe” (participant 4); “senses are magnified,” “I vividly feel my mouth” (participant 5).

These processes also impacted on the physiological manifestations of MPA, which were perceived by the students to be mitigated during instrumental practice and pre-performance in some of the concerts they performed: “I am more aware of how my body responds in potentially anxious performance situations, and I feel I could be more present in the moments before and during a performance” (participant 1). The exploration of enhanced bodily awareness through the implementation of body-centered meditations has been previously investigated in studies not situated within a musical context (Singer and Engert, 2019). The present study aligns with those preceding findings, specifically concerning its application within a musical domain.

The psychological manifestations of MPA, confusion and mental rumination, were also reported to be diminished by participants due to increased mental clarity and positive mental states: “I feel refreshed, with a clear mind,” “I felt pretty focused,” “I felt much more present during recent days” (participant 2); “I am focusing on the present moment” (participant 3); “I feel like my brain is clear” (participant 5).

A previous study (Butzer et al., 2016) found that musicians participating in an eight-week yoga intervention experienced reduced psychological states of confusion. The impact of non-judgmental awareness and acceptance toward emerging thoughts and emotions has previously been correlated with improved cognitive-emotional regulation (Teper and Inzlicht, 2013). This study corroborates earlier findings by demonstrating the beneficial impact of meditative practice on the reduction of mental rumination and confusion surrounding musical practice and performance, as well as enhancing general cognitive-emotional wellbeing.

#### Theme 3: emotional states and meditation

The emotional states experienced by the participants during the 21-day period were predominantly positive, with the exception of a sense of frustration arising from the challenge of sustaining a daily meditative practice with ease. The emotions most frequently associated with the meditative experience were “joy,” “calm,” “lightness,” “gratitude,” “peace,” “comfort” and “self-compassion.” The latter is worthy of particular attention, as previous studies have revealed an inverse relationship between self-compassion and the psychological manifestations of MPA, like mental rumination (Raes, 2010), and between self-compassion and general MPA (Farley and Kelley, 2023).

Self-compassion was intimately intertwined with a profound sense of communal belonging, characterized by the recognition of shared challenges and sentiments among the participants. These dynamic fostered enhanced levels of openness, leading to in-depth discussions concerning the most impactful aspects of MPA and the most salient negative emotions experienced in relation to the encountered obstacles in musical practice and performance. This can be seen in the following quotes: “I am grateful for where I am and the people around me. I feel the connection between the gratitude I feel for my loved ones and what I am trying to do through my music” (participant 1); “I feel at ease with the group” (participant 2); “now I know I am not alone” (participant 3). This openness in discussion is of crucial importance, as the feeling of common humanity is in contrast to isolation and is one of the fundamental components of compassion (Neff, 2003). Furthermore, this self-compassion deriving from feeling as part of a group composed by musicians facing similar issues, might play a crucial role in overcoming shame, a phenomenon that is predominant in MPA sufferers who find themselves studying and working in highly competitive environments (Paese and Egermann, 2024).

### Conclusion

The present pilot study was conducted with the aim of testing designs and procedures to be used in the subsequent main case study. The study was instrumental in determining the targeting of steps for the aforementioned case study; this includes the identification that longer periods and a more extensive recruitment of participants would be beneficial. The design was implemented with additional data collection methods to reinforce the qualitative research approach, suggesting a more assiduous monitoring of the participants’ experience in addition to the diary method, and stimulating broader reflections through the introduction of open-ended questions at different points in the course. Moreover, the study yielded intriguing insights into the impact of meditation: the participants’ perceived impact suggests that even a brief meditation intervention can be effective in reducing the manifestations of MPA and contribute positively to general wellbeing.

In the following section, we present the main case study, which builds upon the procedures established in the pilot study. While maintaining the same categories of meditation, the specific practices were adjusted. Notably, meditation on the five senses was replaced with meditation on the breath, a mindfulness practice focused on thoughts was introduced, and the compassion meditation included in this study features the RAIN meditation developed by Brach (2020). With regard to the analysis of data and discussion of findings, the following main case study considers emerging themes from the pilot study, with final reflections based also on them.

## Main case study

### Methods

#### Participants

Participants were recruited via email, with invitations sent to music conservatoires and universities in the UK, Europe and the US between June and October 2022. Additionally, the study was publicized on the British Association for Performing Arts and Medicine (BAPAM) website. Those who expressed interest in participating were sent an information sheet detailing the course and the project, including the main tasks, namely participation in an online meditation course spanning 4 weeks, completion of a daily diary and answering open-ended questions on Qualtrics. Of the 10 musicians who began the course, seven completed all the project tasks (women = 6, man = 1, age range = 20–54, mean = 36.2, *SD* = 14.3). All participants reported suffering from MPA and having some experience with meditation, particularly mindfulness and yoga, but only as a personal interest. The main methods employed by the participants to manage MPA were in fact meditations or breathing techniques. The demographic data are displayed in the ensuing table, with particulars of their educational or professional status, and the time spent daily on instrumental or vocal practice (Table 3).

**Table 3:** Participants overview.

Participant	Gender	Ethnicity	Age	Instrument	Current student or professional status	Instrumental daily practice
Sara	Woman	Caucasian	21	Voice	Bachelor student	0–30 min
Steven	Man	Asian	20	Piano	Bachelor student	1–2 h
Laura	Woman	Caucasian	51	Woodwinds	Professional musician	1–2 h
Maria	Woman	Caucasian	27	Piano	Bachelor student	3–5 h
Grace	Woman	Caucasian	54	Strings	Master student	Non-daily practice
Liz	Woman	Caucasian	47	Woodwinds	PhD student	1–2 h
Susan	Woman	Asian	34	Voice, live electronics	Professional musician	Non-daily practice

#### Data collection

The data collection phase preceding the intervention was conducted via Qualtrics. Approximately 1 month prior to the beginning of the course, participants signed a consent form and completed a questionnaire pertaining to their demographic data. At the onset of the course, participants were furnished with diaries to record their daily meditation experiences, which were subsequently collected via email upon conclusion. Alongside the time allocated to meditative practice, participants were invited to share the predominant emotions and highlights of their practice on a daily basis. Following the second week and the conclusion of the course, participants were invited to respond to open-ended questions via Qualtrics, enquiring about the perceived impact of each meditative practice in relation to MPA and its manifestations.

#### Procedures

As for the pilot study, this main case study was conducted online due to the enhanced feasibility within the context of post-pandemic circumstances and the demonstrated suitability of online interventions based on previous research (Ivtzan et al., 2016; Krusche et al., 2013). The meditation course was conducted online by Ruth Phillips, a cellist and mindfulness coach who designed the “Introduction to Mindfulness for Musicians” (see Table 4).

**Table 4:** Meditation types practiced within the “Introduction to mindfulness for musicians.”

Week	Meditation type
1	*Body-scan meditation*: This meditation can be practiced sitting or lying down, focusing the attention on sensations of the body touching the ground, and on individual parts of the body one at time, from the feet to the top of the head (Kabat-Zinn, 1994; Monaghan and Viereck, 2011; Williams and Penman, 2011).
2	*Meditation on the breath*: This meditation type can be described as *Shamata* meditation. It is practiced while sitting with an upright back and is based on the perception of the breath which is used as an anchor to counteract distracting thoughts (Kabat-Zinn, 1994; Williams and Penman, 2011). Attention can be focused on the sensation of the breath entering and leaving the nostrils or on the sensation of the breath filling the belly and the rest of the body.
3	*Meditation on thoughts*: This meditation type can be described as *Vipassana* meditation. The theoretical basis of this technique is the observation of the continuous mental chatter and its transitory nature. *Vipassana* meditation is practiced while sitting with a posture that is characterized by dignity; following this, the natural inclination is to maintain an upright but not rigid posture. The hands can be rested on the legs with the palms facing upwards in an attitude of openness and readiness to receive (Kabat-Zinn, 1994; Monaghan and Viereck, 2011; Williams and Penman, 2011).
4	*Meditation on emotions*: RAIN meditation. This meditation is practiced using four steps of emotional exploration: Recognize what is happening; Allow the experience to be there, just as it is; Investigate with interest and care; Nurture with self-compassion (Brach, 2020).

The structure of each session remained consistent throughout the course, commencing with an informative introduction that established the context for the day’s practice. Subsequently, participants engaged in a reflective exchange, sharing feedback and insights on their progress. The meditation practice was elucidated in exhaustive detail and then practiced, thus affording participants the chance to enhance their comprehension and expertise. Finally, each session concluded with a reflective discussion phase, during which participants were invited to pose questions and consolidate their learning.

In the first meeting, the teacher presented mindfulness meditation as a secular practice and delineated its principal characteristics. Then, body-scan meditation was introduced as a potential technique to be utilized in the preparatory phase preceding a performance. The development of body awareness was pursued through intentional exploration of all sensations, including those of tension and discomfort.

The second meeting focused on the potential of breath as a tool for facilitating the exploration of key concepts such as acceptance, equanimity and connection. The objective was to foster an awareness of the breath as a practice for embracing reality in a non-judgmental manner. The teacher elucidated the manner in which breathing can be employed to achieve composure and stability during performance, facilitating a sense of connection with oneself and others. The technique was explained and practiced with a focus on *exploring* the breath rather than attempting to *controllin*g it. Following the second week, open-ended questions were distributed to all participants via Qualtrics with the aim of eliciting detailed feedback on the meditative experience and the perceived impact on MPA exerted by the two meditations practiced.

The third meeting centered on the concept of the “inner critic” in practice and performance. All participants were encouraged to engage in a process of analysis and differentiation between the concepts and attitudes of compassionate observation and self-judgment, considering their occurrence within the context of their instrumental practice. Indeed, music students frequently exhibit a self-critical attitude and automatic thoughts from the very outset of their musical practice, even when they are merely picking up their instruments and playing a few notes. This attitude is so deeply ingrained that it is no longer even perceived or acknowledged by the music students themselves (Paese and Egermann, 2024). In order to identify and acknowledge these unconscious thoughts, it was proposed a meditation practice specifically focused on thought awareness. All participants were encouraged to practice this form of meditation by identifying the nature of their thoughts (e.g., planning, remembering, or judging), and noticing the potential cognitive defusion that could be achieved through the identification of thoughts, perceiving the space between thoughts, and observing what happens when a particular thought emerges in the daylight of experience over others.

The final meeting addressed the topic of emotions and the potential role of emotional awareness in mitigating MPA. The RAIN meditation technique (Brach, 2020) was introduced and practiced with the objective of welcoming emotions as gateways to flow, presence, and creativity. The RAIN meditation technique comprises four principal stages. The four steps are as follows: R, “recognize what’s going on”; A, “allow the experience to be there, just as it is”; I, “investigate with interest and care”; and N, “nurture with self-compassion.”

All sessions were recorded via Zoom and subsequently shared with the participants. Furthermore, open-ended questions were administered via Qualtrics to elicit detailed feedback on the experience and the impact of the meditations practiced in weeks 3 and 4.

#### Data analysis

A twofold approach of inductive and deductive thematic analysis was considered the most appropriate to explore the experience of meditating musicians, the perceived impact of meditations on MPA, and to examine the processes of cognitive and emotional self-regulation in relation to MPA (Braun and Clarke, 2006, 2021; Williamon et al., 2021).

**Figure 1:**
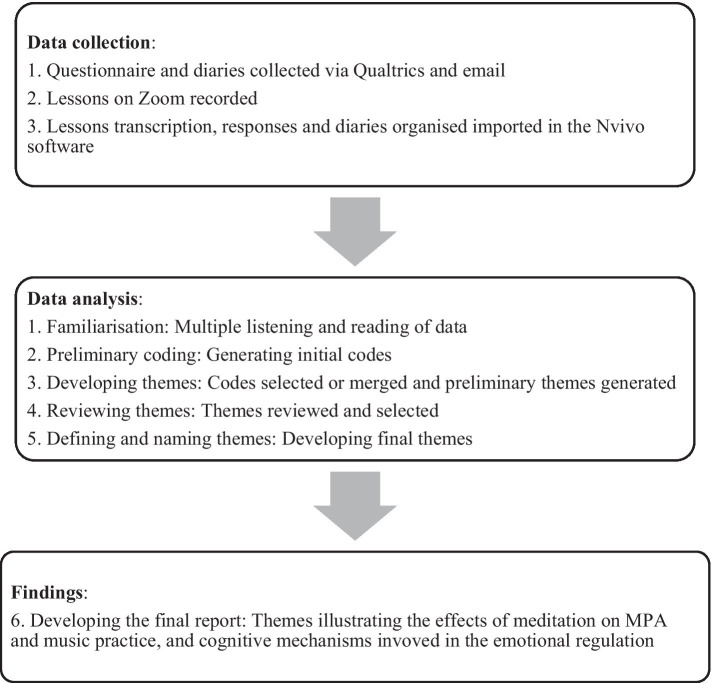
Overview of data collection and analysis procedures.

The analytical procedure was conducted in accordance with a six-step process applied to three qualitative data sets (see Figure 1): diaries, open-ended responses, lessons recordings. The data coding process was conducted with the support of the NVivo 12 software. To facilitate the familiarization with the data, multiple readings of the diaries and open-ended responses were conducted. Similarly, multiple listening and subsequent transcription enabled the researchers to gain a comprehensive understanding of the recorded lessons. Throughout the coding process, the researchers engaged in reflexivity, taking note of personal experiences or biases that could influence the coding process.

While personal experiences played a significant role in interpreting the data, the authors remained critically aware of their position as researchers, continually reflecting on potential biases and ensuring that interpretations were grounded in the participants’ perspectives. This reflexive approach enabled the authors to navigate the complexities of subjective interpretation while striving to maintain rigor, transparency, and validity throughout the data analysis.

The aim of this study was to explore the participants’ experiences with meditation, the impact of those meditations, and the process of emotional regulation occurring by practicing meditation.

Therefore, two phases of analysis were developed consequently:

1. An inductive cross-case analysis was conducted on the entire data set in order to gain insight into their collective experience.
2. A deductive cross-case analysis conducted on the whole data set of all participants with the aim of investigating the collective experience through the lens of cognitive and emotional mechanisms occurring with meditation identified by Dahl et al. (2015).

The codes generated from the open-ended responses, diaries, and participants contributions from lesson recordings were further analyzed to detect similarities and contrasts for generating main themes. In the fourth and fifth phases, the themes were subjected to a process of review and definition, after which they were set out. The final themes resulting from the inductive cross-case analysis are as follows: (1) Instrumental practice; (2) Music performance; (3) Wellbeing. These themes were further explored with a deductive analysis aligned with the cognitive and emotional processes associated with different meditative practices, as previously theorized in the existing literature (Dahl et al., 2015): interoceptive awareness, self-inquiry, acceptance, meta-awareness and meta-cognition, experiential and cognitive defusion, cognitive reappraisal, and perspective change.

### Findings and discussion

This section presents the findings from the inductive thematic cross-case analysis and the deductive thematic cross-case analysis viewed through the lens of the cognitive-emotional mechanisms previously identified by Dahl et al. (2015). The latter framework consists of a three-dimensional classification derived from cognitive science and clinical psychology (see Table 5). The attentional category includes meditations that regulate attention, disengage from distractors, and reallocate attention to the appropriate object. The constructive category includes meditations that promote positive relationships and self-awareness, such as compassion and loving-kindness meditations. These practices have been shown to be an effective intervention for people with social anxiety. The deconstructive category addresses maladaptive thoughts and behaviors through processes of self-analysis, self-inquiry and self-awareness. It includes meditations that facilitate insight, such as Vipassana Theravada.

**Table 5:** Summary of the framework proposed by Dahl et al. (2015).

Meditation practices	Main features of meditation practices	Cognitive mechanisms of meditation practices
Attentional family	Enhance the capacity to maintain a stable awareness of thoughts, behaviors, emotions and perceptions	Meta-awareness Experiential defusion
Constructive family	Cultivate virtuous qualities (e.g. kindness, compassion)	Meta-awareness Cognitive reappraisal Perspective taking Self-schema
Deconstructive family	Change unhelpful cognitive patterns by gaining insight into the essence and mechanisms of conscious experience	Self-inquiry Insight

#### The inductive cross-case thematic analysis

The three themes presented illustrate the collective experience of musicians who participated in the introductory mindfulness course for musicians and the principal impact of meditative practices (see Table 5). It is important to note that all participants encountered several obstacles in pursuing the meditative practice. These included a tendency toward mental rumination and distraction, as well as logistical challenges in identifying a suitable space for meditation. This observation is significant because it underscores the necessity to develop additional support strategies for music students and professional musicians starting a meditative practice.

##### Theme 1: instrumental practice

The implementation of the body-scan and breath meditations has been observed to result in the development of a more effective instrumental practice. In particular, an increased body awareness resulted in a more functional technical change in breath management for Laura (flutist), who also managed to release previously unperceived muscle tensions: “I took a slow relaxed intake of breath and only what I would need. I focused my awareness on my body and particularly my feet to feel grounded below me.” Moreover, she was able to include uncomfortable and unpleasant perceptions, as reported in the subsequent sentence:

I noticed that playing felt quite burdensome. Within half a minute I began feeling like I needed a cup of coffee or some sugar sugary food. Playing felt tiring exhausting. The feeling I had was that this is really hard and what I was referring to be the constant work involved every day in maintaining a good sound. (Laura).

Difficult emotional states emerging with meditation were perceived, contained, and included. Indeed, in reflecting on her approach to instrumental practice, she also became aware of a narrative that emphasized the challenges and perceived difficulties associated with instrumental study. While this may initially seem discouraging, it does, however, represent a potential first step toward developing the awareness needed to challenge this narrative and overcome perfectionism.

I became aware that when I do my long notes to practice my tone work, I have a narrative that it is hard work, exhausting and boring and that it is really hard to get and maintain a good sound on the flute. I really do not get any further than long notes at the moment each day because I feel so discouraged with the amount of work required to get a good tone. I have been playing for many years, and I think other players just get on with the daily long note practice but the constant battle to maintain a good sound just discourages and exhausts me (Laura).

Laura also noted that the time dedicated to meditative practice had not yet had an impact on her perspective toward musical practice and performance.

There has been no change in how drained and discouraged I feel though. I heard one of the other participants say that the experience made her feel like her instrument was her friend. I do not feel like that at all, none whatsoever, but at least I was able to be aware of it after I’d done the body scan and went to play (Laura).

The integration of positive and negative emotional states represents a crucial element in the development of a comprehensive wellness model, as evidenced by the recent focus on ambivalent emotions, which are a prominent feature of the meditative experience (Lomas, 2019). This was a particularly sensitive aspect for Laura, which would probably require a longer period of meditative practice specifically directed toward this cognitive-emotional aspects.

In the case of Sara (singer), a better breath control occurred: “It’s strange, because I always control my breath.” Moreover, she reported amplified body sensations and expressed enthusiasm for the vividness of her perceptions: “Magic, I felt that my hair was breathing, and the breath was in the whole body!.” A comparable impact of mindfulness practice on music students has been evidenced by Czajkowski et al. (2020), with the acquired awareness of specific body parts.

It is noteworthy that body-centered meditations also demonstrated an impact on cognitive anxiety manifestations, namely a reduction in mental rumination. Grace (violinist) reported a positive impact of body-scan meditation on her response to notational errors. These errors were no longer met with excessive criticism or mental rumination. She reported a significant impact on cognitive aspects and the benefit that awareness of one’s own cognitive processes can bring in terms of self-regulation: “The body-scan meditation making me notice, with less angst, the degree to which thoughts intrude, and the awareness that I can nudge them away before they become fully fledged.” The meditation on thoughts proved to be the most impactful for Grace. It led to an immediate realization of how to mitigate the cognitive-emotional component of music practice.

Accepting that thoughts come through as I’m playing but treating them like clouds that pass through rather than “feeding” them with attention. Yes, I would still cock up a note because of them, but I could move on immediately so avoided the cascading feeling of failure and shame (Grace).

This testimony illuminated two particularly distressing experiences associated with MPA, the feelings of failure and shame, which frequently act as additional emotional burdens, and intensify the overall impact of the experience. In this regard, recent studies have indicated that women are more prone to experiencing guilt and shame, and that these two factors are predictive of stress and MPA (Coşkun-Şentürk and Çırakoğlu, 2018).

Through the practice of meditation on thoughts, Liz (flutist) was able to gain a deeper understanding of the relationship between her thoughts and mental rumination during the instrumental practice. She discovered that by learning to let thoughts flow without resisting them, she was able to prevent exhaustion.

Meditation created space in my head, showing myself greater kindness by not engaging as much with harsh critical voices. I am aware of the critical voices in my head and accepting they are there, but I do not need to believe they are speaking the truth (Liz).

This aspect proved to be the most challenging for her to navigate, both in the context of musical performance and in her broader life.

##### Theme 2: music performance

The perceived positive impact on MPA was associated with specific meditative techniques by the participants. The development of body awareness and the acceptance of unpleasant sensations, particularly through the practice of body-scan meditation, have emerged as fundamental factors in the mitigation of MPA manifestations, like in the experience reported by Sara (singer): “It’s strange, because I always control my breath.” Moreover, she was able to overcome voice trembling and achieve greater stability during a musical performance: “Just a moment before the show I was sweating and my voice was tremulous, then I remembered to breathe, and the tremor stopped.” Following a period of just 2 weeks, Sara was able to report a reduction in somatic MPA. These findings align with those of previous studies that have demonstrated the efficacy of meditation in reducing MPA (Chang et al., 2003; Czajkowski et al., 2020). Furthermore, they provide additional evidence of the beneficial impact observed after 2 weeks of practice.

Grace (violinist) effectively addressed and resolved her challenges with mental rumination, resulting in improved inner and outer perception.

I have specifically thought about gravity and grounding before playing. It’s a strange thing to have to, on the one side, listen to one’s inner and outer world; but also, then try to be in the flow when performing. How to turn, the dimmer switch down on the first to enable the second (Grace).

While performing, Grace was able to observe the tendency for muscle stiffness and counteract it with meditative practice. This observed impact was particularly related to the meditation on the breath: “Focus on breathing has always been a useful tool for me.” She reported not only enhanced capacity for a cognitive-emotional self-regulation but also a renewed enthusiasm for playing for others. The latter beneficial impact was previously demonstrated in the study conducted by Chang et al. (2003) on 19 music students who engaged in meditation for 8 weeks and reported increased pleasure and relaxation before the performance. The findings from this study indicate that meditation has a positive impact on the experience of positive emotions during performance, even after a shorter period of practice.

I had an orchestra performance, thoughts came as usual, voices that takes me out, but this time I did not push them vigorously, I just did not pay any attention. I had not enjoyed performing from so much time! I loved playing this way. The less attention I gave them, the best I could play. It was fabulous (Grace).

She did not experience overwhelming shame and criticism with regard to the notational errors. Moreover, she reported a strengthened connection with the audience, and was gratified by her musical performance, which she had not experienced for a considerable period of time.

The practice of the RAIN meditation adapted for musicians, has resulted in an enhanced sense of self-compassion and appreciation for the strength required to embrace vulnerability, a quality of particular significance within the context of a musical career.

With this unusual job that we have, we are literally putting ourselves naked in front of people to be judged. That vulnerability is extraordinary and incredibly brave, because not only you are supposed to give them enjoyment but also people will always have different opinions or have something negative to say. So, it is phenomenally brave to do this, and we are looking on how to make it easier (Grace).

Previous studies in non-musical context have already demonstrated that affect-centered meditations have resulted in increased self-compassion and the development of compassion-based qualities among non-musicians (Singer and Engert, 2019). The findings of this study corroborate earlier research in a musical context.

Concerning the practice of RAIN meditation, the main observed impact for Susan (singer, live electronics), was enhanced resilience accompanied by a notable decline in the behavioral manifestations of her MPA. This has led to an increase in the number of auditions that she would previously have avoided: “I’ve auditioned a lot more for different things afterwards. I was still avoidant, but I got through it anyway.” Susan’s experience highlighted a pervasive issue among professional musicians, as evidenced by her continued engagement in 20 to 60 musical performances annually despite suffering MPA. Indeed, previous research has demonstrated that not only students who have perhaps underdeveloped strategies for coping with MPA are affected by it, but also professionals with established musical careers (Steptoe, 1989). The persistent pursuit of strategies to mitigate MPA may not necessarily indicate an imminent resolution but could potentially exacerbate the overall state of health and induce fatigue associated with the demands of a musical profession, rather than fostering positive and fulfilling emotional states. This aspect represents a significant rationale for the continued necessity of research and the development of more effective tools for the enhancement of musicians’ wellbeing and the amelioration of the more debilitating manifestations of MPA.

Steven (pianist) observed the persistence of somatic manifestations of MPA yet noted an enhancement in self-confidence: “On the physiological aspect it is still a problem, and I often fail to control it, but at least I can think positively about the future performance without judging myself too much.” This may be possibly compatible with the brief duration of the course, indicating that with continued practice over time, even those physiological manifestations that still present a challenge in piano performance may subside, as previous research evidenced (Chang et al., 2003; Czajkowski et al., 2020; Khalsa et al., 2009). It is also important to note that Steven has deviated from the established meditative practice, which limits the ability to determine whether this persistence of somatic MPA was due to misdirection.

##### Theme 3: wellbeing

A number of musicians have attested to the beneficial effects of meditative practice on various aspects of their lives and health (Sara, Steven, Maria, Liz, Susan). One aspect that was considered important by the subjects is an increase in bodily awareness, particularly of physical pain and thoughts. In her account, Liz indicated an ability to discern her physical discomfort; the ability to maintain relaxation while perceiving physical discomfort has been documented on several occasions in her diary: “Relaxed but aware of physical discomfort,” “aware of physical aches and pains, am relaxed though.” Steven demonstrated an aptness for maintaining composure amidst the turbulence of his thoughts, particularly in terms of reduced self-criticism and enhanced confidence. These findings are consistent with previous non-musical research, which demonstrated the development of compassion-based qualities through the practice of meditations on thoughts and emotions (Singer and Engert, 2019).

Similarly, Maria exhibited a capacity to mitigate the impact of mental ruminations through an anchoring in her physical sensations and an orientation to the present moment: “I appreciated learning something more about myself, like understanding how to take control of my body and my thoughts, even if it can be more complicated than it seems.”

Furthermore, meditation has been shown to facilitate emotional regulation in challenging circumstances, promoting a more compassionate attitude toward oneself. The practice of meditation on the breath proved an effective method for Susan in managing challenging emotions associated with relationships and insomnia: “I suffer from insomnia and had a tough conversation. I breath and I could feel emotions and thoughts without reacting.” Therefore, meditation has been helpful in improving emotional regulation in difficult times, with a more compassionate approach to themselves. This finding corroborates prior qualitative research that demonstrated the efficacy of mindfulness in navigating intense emotions in relationships among musicians (Czajkowski, 2018).

Similarly, Liz demonstrated an increased capacity for compassion, recognizing that she was exerting her utmost efforts in navigating the demands of familial and occupational responsibilities: “busy, jolly, coping with family situations and work deadlines,” and experiencing stress, though not to an excessive degree. Sara conducted a more comprehensive exploration of her inner self, which contributed to an increase in both self-confidence and relaxation, which she attributed to both her experience with the practice and her sense of connection with the group. The primary emotional response she experienced was fear, which she attempted to integrate and acknowledge: “There was fear, but I tried not escape from it” (Sara). Furthermore, the final week of meditation was characterized by a sense of cognitive and emotional alignment and integrity: “My body was in tune with my mind” (Sara).

The participants of this study exhibited enhanced capacity to accept feelings, thoughts, and emotions, which contributed to more effective cognitive and emotional self-regulation. The most commonly reported cognitive impact was a reduction in mental ruminations and an increase in attention span. This impact is consistent with previous non-musical quantitative research that emphasized the effects of meditation on cognitive processes, including enhanced focus and presence, increased awareness of internal thoughts and a shift toward positive emotional states (Singer and Engert, 2019). The primary emotional effect was a decrease in self-critical thinking (Liz, Sara, Maria, Grace).

Regarding the most beneficial effect perceived by this meditation practice, I think it’s hearing the critical voice and moving past it anyway instead of latching on to each critical thought. I was reminded that these are voices from the past that got installed in my brain and I get to choose to engage with them or not most of the time (Susan).

In this contribution, Susan elucidated a topic that has been extensively explored in the domain of psychological therapy (Mongrain, 1998). Self-critical thoughts are the result of the internalization of criticism or expectations perceived at an early age by parents or caregivers, and subsequently internalized as inner judgmental attitude (Mongrain, 1998). A shift in this attitude is achieved by disengaging from those self-limiting and judgmental thoughts, recognizing them as an external phenomenon from which one can disidentify (Juncos and de Paiva e Pona, 2018).

The potential for sharing emotions and thoughts within a group context was identified as a highly beneficial factor in mitigating feelings of fear of judgment from others, as well as feelings of loneliness: “It was also a great pleasure to meet people who share similar emotions and thoughts in certain circumstances. Now I know I am not alone” (Maria); “I felt a relief that other people experience really similar issues, and community in sharing our experiences” (Liz).

With respect to impact on mood states, an improvement was observed in positive emotional states (Sara, Steven, Maria, Grace, Liz, Susan), awareness and acceptance of difficult emotions (Sara, Steven, Laura, Maria, Grace, Liz, Susan), and awareness and elaboration of ambivalent emotions (Sara, Laura, Grace, Liz).

#### Deductive thematic analysis

The deductive cross-case analysis was conducted with the objective of identifying the cognitive mechanisms occurring with each meditative practice. This was achieved by analyzing the themes developed with the inductive cross-case analysis (see Table 6) through the framework developed by Dahl et al. (2015) applied to each meditative practice included in this case study (see Table 5).

**Table 6:** Themes and subthemes.

Instrumental practice *n* = 4	Music performance *n* = 4	Wellbeing *n* = 7
Improved body awareness due to body-centered meditations Improved breath management More functional instrumental technique Muscular tensions mitigation Inclusion of ambivalent emotions Reduced mental rumination and self-criticism for notational errors	Reduced cognitive MPA Reduced somatic MPA Increased body awareness Increased contextual inner and outer awareness Reduced self-criticism Increased compassion Self-regulation of emotions and thoughts	Increased body awareness Positive mood states Prosocial behavior Emotion self-regulation Ambivalent emotions Awareness of unhealthy habits Increased self-compassion Reduced mental rumination

This analysis represents a preliminary stage in the investigation of the constitutive mechanisms of individual meditations, which may act as mediators between meditation and the perceived impact. Among these mechanisms, interoceptive awareness and acceptance are common to all meditative practices, and fundamental to other mechanisms which do not follow a linear process but rather a circular one. In fact, by addressing physiology, thoughts and emotions in order, those mechanisms occurred several times in the weekly lesson as well as in daily practice.

##### Body-scan and meditation on the breath

The cognitive processes involved in meditation, as identified by Dahl et al. (2015), indicate that both body-scan and breath meditations are considered attentional meditations, aimed at strengthening self-regulatory processes of attention and meta-awareness of emotional, cognitive, and physiological processes. The aforementioned research indicates that practicing these meditations results in an extended and heightened attentional capacity, accompanied by the development of enhanced body awareness. The participants’ reports have revealed that meditation exerts a beneficial influence on cognitive and emotional regulation, thereby mitigating MPA (see Table 6).

The participants’ perspectives reported in the qualitative data presented in the previous section, suggest that the development of body awareness through body-scan and breath meditations represented a means of counteracting the physiological and cognitive manifestations of MPA. This was perceived to enhance wellbeing by fostering a sense of security in the body and breath as safe anchors. Additionally, the practice was found to facilitate the development of instrumental study strategies and instrumental sound. The findings of this study corroborate those of previous research in the field of contemplative sciences, which identify body interoceptive awareness as a pivotal component of mindfulness protocols. This is because the lack of sufficient body and mind sensitivity can result in a decline in physical and mental wellbeing (Kabat-Zinn, 2003; Farb et al., 2015). Furthermore, the findings are consistent with those of earlier studies on the beneficial impact of non-judgmental body awareness cultivated through somatic and mindfulness approaches on instrumental sound quality (Dora et al., 2019) and practice efficiency (Czajkowski et al., 2020).

The perceived positive impact of developing body interoceptive awareness was experienced alongside acceptance, which is a core dimension of mindfulness (Cardaciotto et al., 2008). It can be argued that the absence of acceptance may render the increased body awareness less beneficial. The participants indicated that acceptance and the ability to remain non-reactive to unpleasant physical sensations were beneficial for enhancing both musical performance and general health. This finding aligns with previous research on the efficacy of mindfulness techniques in the pre-performance period, which has demonstrated the capacity to enhance attentional focus and modulate emotional responses (Kaleńska-Rodzaj, 2020). The beneficial effects of meditative practice extended beyond the alleviation of the physiological and psychological symptoms associated with MPA. Additionally, it led to an enhanced connection with the audience and an increased sense of presence in the moment, both of which are crucial for the optimal expression of a musician’s abilities (see Table 6). The mechanism of interoceptive awareness has expanded from an awareness and acceptance of the body to a meta-awareness of perceptual processes. Meta-awareness is defined as “the ability to monitor non-focal aspects of experience (e.g., feelings, thoughts) while focusing on a goal or object” (Diaz, 2018). A positive impact was observed with regard to the instrumental practice, which led to an increase in the capacity for non-judgmental self-critical observation and attention, as well as the fostering of more effective study strategies.

The practice of these mindfulness meditations has been reported by participants as a genuine means of overcoming the spiral of MPA symptoms when meta-awareness resulted in experiential defusion. The results demonstrated a meaningful effect from cognitive defusion in counteracting automatisms, which is consistent with previous research on the effects of mindfulness on instrumental practice (Czajkowski et al., 2020).

##### Meditation on thoughts

**Figure 2:**
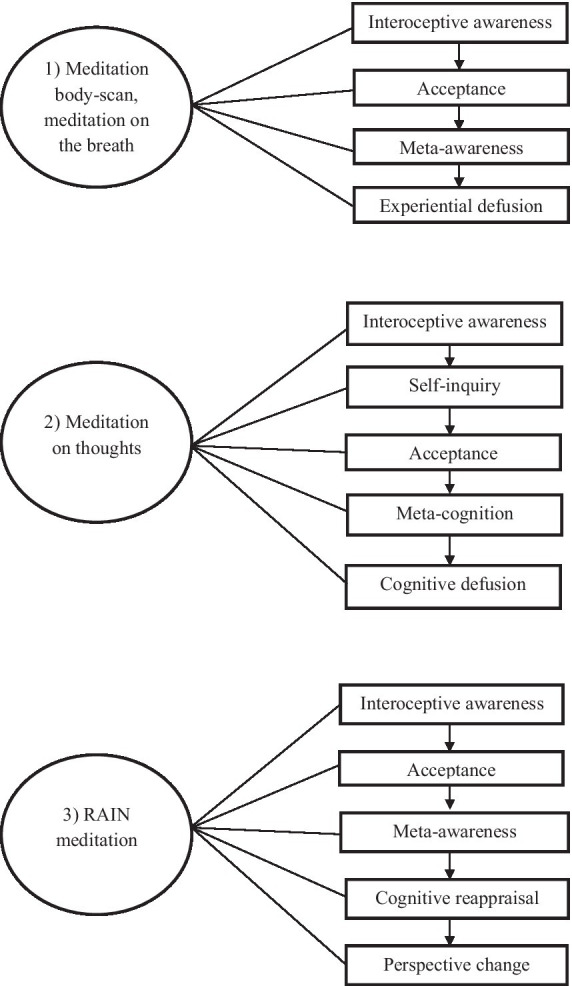
Cognitive processes of “Introduction to mindfulness for musicians”: (1) meditation body-scan, meditation on the breath; (2) meditation on thoughts; (3) RAIN meditation.

The processes involved in the meditation on thoughts include the mechanism of interoceptive awareness as a foundational basis, as with other meditations (see Figure 2). Together with self-inquiry, mindfulness about thoughts enabled musicians to develop conscious perception of cognitive processes, in line with previous research on this type of meditation (Dahl et al., 2015). The discernment of the nature and effects of unhelpful or unhelpful thoughts on health, instrumental practice and musical performance has outlined a direction for dealing with them effectively.

The ability to distinguish between useful and unhelpful thoughts has been identified as a crucial factor in regulating cognitive and emotional processes in daily life and during instrumental practice. By recognizing and accepting that some thoughts are not useful, individuals can reduce mental rumination and enhance their attentional capacity. Furthermore, participants reported the negative impact of self-critical thoughts resulted in somatic manifestations, which demonstrated the physiological consequences of dysfunctional thoughts and emotions. This finding is consistent with previous non-musical research (Porges, 2001).

The process of cognitive defusion was achieved through metacognition, which resulted in a state of cognitive disengagement from negative thoughts. This approach proved to be beneficial in the context of overcoming the negative spiral of thoughts that can affect an individual’s performance in music, as well as in terms of their overall wellbeing. This effect is consistent with previous research, which has identified “de-reification” as a fundamental psychological process implemented with mindfulness protocols. De-reification refers to the ability to identify and let go of thoughts by understanding that they are merely thoughts (Lutz et al., 2008).

##### RAIN meditation

RAIN (Brach, 2020) is an acronym that illustrates the steps involved in this meditation process with regard to emotions: recognize, allow, investigate and nurture. Prior research has identified perspective-taking and cognitive reappraisal as the constitutive mechanisms of this type of meditation, which serves as a means of cultivating values and mindsets that enhance personal and social emotional wellbeing (Dahl et al., 2015). The preliminary model for the emotional regulation process identifies interoceptive awareness as the foundational step (see Figure 2). This step is considered in conjunction with acceptance and meta-awareness, as well as other meditations, to ascertain its meaningful perceived effects.

The observed foundational importance of interoceptive awareness is consistent with previous research indicating that enhanced differentiation of emotions is associated with improved emotion regulation (Barrett et al., 2001). Following the development of conscious perception of emotional processes, the mechanism of cognitive reappraisal was identified as a key element within the emotional regulation process facilitated by this meditation. Previous research findings (Gross, 2001) have also indicated that reappraisal is an effective strategy for emotional regulation. For instance, Brooks (2014) proposed that positively reappraising anxiety as excitement can lead to enhanced performance in the pre-performance context. The conclusive mechanism potentially leading to positive effects on MPA reduction was identified as perspective change, with situations and people perceived as pleasant instead of threatening. The positive relational impact with reduced fear of judgment of others and reduced sense of isolation reflects the quantitative data showing a reduction in negative emotional states for six out of seven participants.

The potential benefits of this meditation type have been demonstrated in non-musical contexts, with research indicating positive effects on psychological wellbeing (Neff, 2003; Neff and Germer, 2017). The findings of this study are consistent with those of previous quantitative research in the extra-musical context (Singer and Engert, 2019), qualitative research in the musical context (Paese and Egermann, 2024), and recent developments in performance science, which have proposed that self-compassion may serve as an effective “antidote to performers’ distress” (Walton et al., 2022, p. 78).

In view of the findings, it is of interest to observe similarities between certain aspects of these meditation interventions and cognitive therapy. Mindfulness-based cognitive-behavioral therapies, like Acceptance and Commitment Therapy (ACT, Juncos and Markman, 2016; Strosahl et al., 2017), encompass elements of awareness, acceptance, cognitive defusion, perspective change, and the formulation of actions aligned with meaningful personal values. Furthermore, these approaches to addressing MPA can be considered comparable in that they do not merely seek to reduce symptoms through allopathic means but rather have the holistic objective of integrating the MPA experience with non-judgmental acceptance and enhancing emotional and psychological flexibility.

With regard to the positive impact of mindfulness meditation-based interventions, studies have demonstrated that the MBSR protocol, when adapted for use by musicians, has been effective in reducing MPA, improving aspects of learning and instrumental practice, and enhancing general wellbeing (Czajkowski et al., 2020). Similarly, ACT has been shown to have positive effects in this context (Clarke et al., 2020; Juncos and Markman, 2016).

## General conclusion

The aim of the main case study and its pilot was to explore the impact of a short introductory mindfulness course for musicians suffering from MPA, building on the preliminary findings of a pilot study. The present study contributes to recent scholarship on the value of meditative practice over limited periods (Jayawardene et al., 2017), with findings suggesting that a four-week program can positively impact wellbeing, emotional states, and the manifestation of MPA both before and during musical performance.

The greatest impact on the reduction of MPA manifestations was observed in the case of body-centered meditations, which were found to activate body awareness, thereby enabling the recognition and management of anxious physiological reactions, rather than their overwhelming. Furthermore, the increased body awareness facilitated technical improvements and enhanced attention, which benefited instrumental practice. The awareness of thoughts and the succession of acceptance and defusion had a positive effect on both musical practice and performance, with a decrease in self-critical and perfectionist attitudes, particularly in relation to notational errors. The process of awareness and acceptance of challenging or ambivalent emotions through RAIN meditation has yielded favorable outcomes with respect to relationship management and prosocial behaviors through group sharing.

These findings are consistent with those of previous research on the beneficial effects of meditation in reducing MPA and enhancing wellbeing (Chang et al., 2003; Czajkowski et al., 2020; Khalsa et al., 2009). Additionally, the impact of individual practices aligns with the findings of research conducted on the effects of specific meditations in a non-musical context (Singer and Engert, 2019). The persistence of somatic and cognitive manifestations of MPA for two of the participants, with a more challenging personal history and a greater need for work on attentional states, suggested that although the brief duration of meditative practice may yield effective benefits, it is still to be considered as an initial step toward a longer-term commitment that may lead even those with greater obstacles to overcome to beneficial results.

The online format was found to be suitable for the purposes of this study, as has been highlighted by previous research (Ivtzan et al., 2016; Krusche et al., 2013), and it may also be repurposed due to the convenience of using the online content at a distance. Further investigation is needed to determine the impact of individual meditative practices, and this study has only explored the impact of a limited number of those with potential for use as MPA management tools. Therefore, it is to be hoped that there will be subsequent qualitative research, which is still limited in relation to this research subject (Barros et al., 2022), aimed at investigating which practices are most appropriate for use in mitigating specific manifestations of MPA, differentiating their use for physiological, cognitive-emotional and behavioral manifestations.

In terms of limitations, it should be noted that this study did not include a musical performance. It would undoubtedly have been beneficial to be able to examine participants’ experiences before, during, and after a music performance in order to investigate the impact of meditative practice on MPA. It would therefore be beneficial for future studies to incorporate a music performance, both before and after meditative practice, in order to gain a more comprehensive understanding of the perceived impact of meditative practice on musicians’ ability to perform without being limited by the manifestations of MPA.

This study contributed to the disentanglement of the effects of meditations on MPA, in addition to investigating the various cognitive mechanisms involved in individual meditations. The findings may be of interest to musicians who employ meditation as a means of emotional self-regulation and coping with MPA, thereby encouraging the appropriate and informed use of meditative techniques. Similarly, this study may be of interest to educational institutions that are increasingly concerned with the wellbeing of their students and the nurturing and development of the emotional skills that contribute to their becoming fulfilled professional musicians.

## Data Availability

The raw data supporting the conclusions of this article will be made available by the authors, without undue reservation.
